# Risk of occupational infection in forensic workers: a review

**DOI:** 10.1093/fsr/owad001

**Published:** 2023-01-13

**Authors:** Laurène Dufayet, Jerome Langrand, Bertrand Ludes

**Affiliations:** Unité Médico-Judiciaire, APHP - Hôpital Hôtel Dieu, 75004, Paris, France; Centre AntiPoison de Paris, Fédération de Toxicologie, APHP - Hôpital Fernand Widal, 75010, Paris, France; Inserm UMRS-1144, Faculté de Pharmacie, 75006, Paris, France; Université de Paris-CNRS UMR 8045 Babel, 75005, Paris, France; Centre AntiPoison de Paris, Fédération de Toxicologie, APHP - Hôpital Fernand Widal, 75010, Paris, France; Université de Paris-CNRS UMR 8045 Babel, 75005, Paris, France; Institut Médico-Légal, 75012, Paris, France

**Keywords:** forensic sciences, occupational risk, forensic worker, review, infectious risk

## Abstract

The occupational risk of infection in forensic workers is a cause for concern, furthermore in the current context of the coronavirus disease-19 (COVID-19) pandemic. In order to characterize this risk, we performed an extended review of the literature on occupational infections occurring in forensic workers. Seventeen articles were included. Direct contamination by aerosolization was the main mode of transmission reported, with 17 cases of tuberculosis. Indirect contamination was described as the mode of transmission in 10 cases (five cases of blastomycosis, two cases of tuberculosis, two *Streptococcus pyogenes*, and one case of human immunodeficiency virus). In all the other included cases, the mode of transmission was unknown. For two of them, the information provided was sufficient to link them to occupational exposure (one case of toxoplasmosis, one case of tuberculosis). For the remaining 10 cases, the link was uncertain (six cases of tuberculosis, three of hepatitis B, and one of COVID-19). Even if there is probably significant under-declaration, the number of infections linked to an occupational risk in forensic workers is not alarming, thanks to effective preventive measures.

## Introduction

Occupational risk in forensic workers is a cause for concern, as pathologists and autopsy technicians are subjected to many health and safety risks, including the risk of infection. Many famous physicians have, in the past, contracted infections during cadaveric dissection [1]. This is the case, for example, of Marie François Xavier Bichat (1771–1802), a famous French surgeon who died from hemoptysis after a long history of tubercular infection, that he most likely contracted during dissection [1]. Since then, hygiene and prevention measures have evolved considerably, but the infectious risk is still present. In fact, studies have shown that postmortem room staff had amongst the highest rates of laboratory acquired infection in British clinical laboratories [2]. Most pathogens have long survival times outside the body, or on a dead body preserved at cold temperatures. This is the case for instance for human immunodeficiency virus (HIV, 16 days), certain mycobacteria (8 weeks), or coronavirus disease-19 (COVID-19, 5–9 days) [3–6]. With the COVID-19 outbreak and the heightened infectious risk, some centres even replaced traditional autopsy with postmortem computed tomography due to concerns over infections [7]. This work endeavoured to characterize the actuality of the infectious risk in autopsy, based on an extended review of the literature on occupational infections occurring in forensic workers.

## Methods

We searched the PubMed database with the following Medical Subject Headings (MeSH) terms “occupational”, “infection”, “injuries”, “autopsy”, and “postmortem”. The database search was performed on publications released between January 1950 and November 2021. Article selection was performed through several levels of study screening. A primary screening for titles of the reports was done by one investigator to exclude irrelevant articles. Exclusion criteria were as followed: articles not written in English or in French, articles referring to veterinary practice, articles referring to deaths from occupational causes. On secondary screening, two investigators reviewed the abstracts and the full texts of all the remaining articles. All case reports of contamination were included. For other types of articles, mostly guidelines of good practices, they were excluded if they did not mention any new occupational contamination. If a contamination was mentioned, they were analysed to extract information about the case (type of biological agent and mode of exposure). We also examined all references from full text articles and included new articles. Articles were checked to exclude redundant cases. All information about documented contaminations was then compiled in a table.

## Results

A total of 45 relevant articles were identified, published between 1972 and 2021. Among these, nine were excluded as they were not written in English nor in French, two because there was no abstract or full text available. Thirty-four publications remained. Twenty articles were excluded from our analysis because they did not mention any new case of occupational contamination [8–27]. These were mainly recommendations for good practices in high-risk autopsies or literature reviews that did not present any new case. Fourteen articles were included, and are presented in Table 1 [5, 28–40]. Examining all references from full text articles allowed us to include three more articles [2, 41, 42]. A total of 17 articles was included, and they were checked for redundancies, as some appear to describe the same HIV contamination case [28, 29].

**Table 1:** Published cases of occupational acquired infections among forensic workers.

References	Pathogenic agent	Number of cases/route of exposure	Other information
[5]	COVID-19	1/unknown	Unclear mode of contamination (occupational or private).
[29]	HIV	1/cutaneous inoculation	Scalpel wound to the hand, probably the same case report in [28].
[28]	HIV	1/unspecified	
[33]	Tuberculosis	1 pulmonary tuberculosis/aerosolization	A 40-year-old with BCG immunization in childhood. Developed pulmonary tuberculosis 1 year after the autopsy of an undiagnosed tuberculosis patient.
[30]	Tuberculosis	7 asymptomatic infections/1 pulmonary tuberculosis/aerosolization	Autopsy of a patient with unsuspected active tuberculosis. Eight out of 35 Mantoux-negative students became infected and one developed the clinical disease.
[34]	Tuberculosis	3 asymptomatic infections/1 pleuritic tuberculosis/aerosolization	Autopsy of a patient with unsuspected active tuberculosis. One case of pleuritic tuberculosis in an autopsy staff member, with BCG immunization in childhood, 5 months after contact.
[35]	Tuberculosis	4 pulmonary tuberculosis/aerosolization	Autopsy of a patient with unsuspected active tuberculosis. All subjects were BCG immunized.
[32]	Tuberculosis	1 “prosector’s wart”/cutaneous inoculation	“Trauma” during autopsy. Only the abstract was available.
[37]	Tuberculosis	1 “prosector’s wart”/cutaneous inoculation with a bone fragment	A 31-year-old male pathology resident, while performing an autopsy on a patient with cavitary pulmonary tuberculosis, pricked the medial aspect of his left middle finger on the sharp point of a rib.
[38]	Tuberculosis	1 “prosector’s wart”/unknown	Only the abstract was available.
[2, 40, 41]	Tuberculosis	6 unspecified cases/unknown	Not enough information to retain occupational infections.
[2]	Hepatitis B	3 unspecified case/unknown	Not enough information to retain occupational infections.
[36]	*Streptococcus pyogenes*, M type 1, T type 1.	1/cutaneous inoculation	Superficial nick in the skin of a finger while assisting at a necropsy examination of a 73-year-old woman who had died from septicemia caused by *S. pyogenes*.
[39]	*S. pyogenes*, Groupe A, β-hemolytic	1/cutaneous inoculation	Scalpel wound during an autopsy, evolved in necrotizing fasciitis. The body undergoing postmortem examination had no history of necrotizing fasciitis, but no tests for group A β-hemolytic *Streptococcus* had been undertaken.
[42]	Toxoplasmosis	1/unknown	A pathologist became acutely ill with toxoplasmosis 2 months after the autopsy of a patient who died with toxoplasmic ventriculitis.
[31]	Blastomycosis	5 (3 of which are described as historical cases)/cutaneous inoculation *via* scalpel wound to the hand	One of the historical cases was treated by amputation of the injured finger.

BCG: Bacillus Calmette-Guerin; COVID-19: coronavirus disease-19.

## Discussion

The risk of an occupational infection depends on several factors: the type of biological agent, the mode of exposure and its degree, and the individual characteristics of the worker, such as his/her immunization status. The availability of efficacious post-exposure prophylaxis should also be accounted for [8]. Many biological agents are present in the autopsy room. They can be the cause of death, with meningococcal or pneumococcal infections for instance [43], but also part of the patient condition. Thus, at the medico-legal institute of Lille (France), Pioche et al. [44] screened 77 consecutive cadavers for hepatitis C virus (HCV) markers. They found HCV RNA in the cadaveric blood of almost 19% of their cases *versus* an HCV seroprevalence of 0.75% in the French population. Of course, the presence of HCV RNA in cadaveric blood does not necessarily entail viable virus, but these results still highlight the high prevalence rate of HCV in medico-legal cases, mostly in intravenous drug users [45]. Infectious agents, mostly bacteria and fungus, can also develop postmortem, but there is little literature on this subject [18, 46]. Moreover, all germs (microorganisms) seem to have a long-lasting postmortem viability. Viable HIV was detected in blood up until 16 days after death [3], mycobacteria endure in sputum up to 8 weeks at 4°C [4] and were even proved to resist formalin fixation [9]. Regarding COVID-19, a study showed that the longest postmortem interval with positive severe acute respiratory syndrome coronavirus 2 (SARS-CoV-2) RT-qPCR on body surfaces was 9 days but that no viable SARS-CoV-2 was found on the skin nor on the body bags of patients deceased from this infection [5].

This is the first article, to our knowledge, to index all occupational infection described in autopsies. The main limitation of this review is of course the under-reporting of occupational infections. This bias is well known in occupational studies, especially in the medical field [47]. As this article is a literature review, we did not question the validity of statements made by the authors of the included articles.

Some cases described as occupational may indeed have been caused by private contamination. Nevertheless, this article showed some interesting findings. Concerning the mode of exposure, infectious contamination can occur in many ways in autopsy. Direct contamination may happen *via* ingestion, inhalation, or projection of contaminated droplets. This risk is well known for tuberculosis, but Johnson and Robinson [48] showed that HIV can be found and cultured from vapours generated by an oscillating bone saw. Indirect contamination can happen from cuts or stings. For these, the scalpel is the first vector that comes to mind but, in autopsy, bone fragments or foreign bodies can also constitute a risk. Hutchins et al. [49] described an additional risk due to retained needle fragments in the bodies of intravenous drug addicts and Abraham and Greenfield [16] alerted on the hazards of vena-cava filters that have a sharp anchoring hook.

In this review, we found 17 cases of occupational infections with direct contamination by aerosolization, all of them concerning tuberculosis [30, 33–35]. For the 10 cases linked to indirect contaminations, eight were caused by scalpel wounds (one case of HIV [28, 29], five cases of blastomycosis [31], and two cases of *Streptococcus pyogenes* [36, 39]), another one was caused by a wound occasioned by a bone fragment (tuberculosis) [37], and the last one was caused by an unspecified trauma (tuberculosis) [32]. In two cases, the route of contamination was unknown but the information was sufficient to link them with occupational exposure (one case of toxoplasmosis [42], one case of tuberculosis [38]). Six cases of tuberculosis [2, 40, 41], three cases of hepatitis B [2], and one case of COVID-19 [5] were included in Table 1, but the links between occupational exposure and the disease were uncertain. A case concerning a histopathology technician was not included but was of interest: she did not work in an autopsy room but for more than 20 years on a daily basis she handled formalin fixed brains and died of Creutzfeldt-Jakob disease (CJD) [50]. Even if the occupational nature of her illness is not proved, this is, to our knowledge, the only published case about CJD that could result from occupational exposure in the postmortem field. Our article shows that most contamination in autopsy results from aerosolization of tuberculosis bacillus, especially when the infection is unsuspected at autopsy. These findings emphasized the importance of preventive measures. They are extensively discussed in many articles [10, 11, 51].

To preserve the health of their workers, autopsy centres should implement three levels of controls. The first one is the implementation of administrative controls to suppress or reduce the risk of exposure in a comprehensive manner. Administrative controls can consist in the implementation of standard safe operating procedures, education of pathologists, and other mortuary workers on their occupational risk, proper equipment maintenance. The second one is the implementation of environmental controls to prevent the aerosolization of pathogens. This should at least include an air-conditioning system in order to assure an adequate air exchange [8]. The last level of controls resides in individual protection measures. We can only recommend wearing suitable respiratory protective equipment (RPE) when taking part in an autopsy, in addition to basic equipment such as protective gloves and glasses. Systematic immunization of the workers for hepatitis B and tuberculosis also seems necessary. Some authors even advise pathologists not to take part in high risk autopsies if they are themselves immunosuppressed [13]. The COVID-19 has proved, if needed, that pathology autopsy and mortuary services are of great importance in the event of a pandemic [52].

Even with the limitations discussed above, this article suggests that the number of cases of occupational infections during autopsy is not alarming. The infectious risk is nevertheless real, particularly through aerosolization and should not be neglected.

## Conclusion

Pathologists are often unaware of the hazards present within a body before the autopsy. As the autopsy population is different from the general one, with many intravenous drug users, infectious risk is particularly high. Even if there is probably significant under-declaration, this article suggests that the number of cases of infection is not alarming, thanks to effective preventive measures. Nevertheless, pathologists should pay particular attention when performing an autopsy on a suspected tuberculosis case.

## Authors' contributions

Laurène Dufayet and Bertrand Ludes conceived and designed the analysis, Laurène Dufayet and Jerome Langrand collected and analyzed the articles, Laurène Dufayet wrote the initial manuscript, Bertrand Ludes and Jerome Langrand read and corrected the manuscript. All authors contributed to the final text and approved it.

## Compliance with ethical standards

Not applicable (literature review).

## Disclosure statement

The authors report no conflict of interest. The authors alone are responsible for the content and writing of the paper. Bertrand Ludes initial holds the position of Associate Editor-in-Chief for *Forensic Sciences Research* and is blinded from reviewing or making decisions for the manuscript.

## Funding

No funding was received.

## References

[1] Shoja MM , Benninger B, Agutter P, et al. A historical perspective: infection from cadaveric dissection from the 18th to 20th centuries. Clin Anat. 2013;26:154–160. 23037893 10.1002/ca.22169

[2] Grist NR , Emslie JA. Association of Clinical Pathologists’ surveys of infection in British clinical laboratories, 1970–1989. J Clin Pathol. 1994;47:391–394. 8027389 10.1136/jcp.47.5.391 PMC502011

[3] Douceron H , Deforges L, Gherardi R, et al. Long-lasting postmortem viability of human immunodeficiency virus: a potential risk in forensic medicine practice. Forensic Sci Int. 1993;60:61–66. 8340039 10.1016/0379-0738(93)90093-p

[4] Rashid Ali MRS , Parameswaran U, William T, et al. A prospective study of mycobacterial viability in refrigerated, unpreserved sputum batched for up to 8 weeks. Int J Tuberc Lung Dis. 2015;19:620–621. 25868033 10.5588/ijtld.14.0938

[5] Schröder AS , Edler C, Ondruschka B, et al. The handling of SARS-CoV-2 associated deaths—infectivity of the body. Forensic Sci Med Pathol. 2021;17:411–418. 34076852 10.1007/s12024-021-00379-9 PMC8170863

[6] Ventura F , Barranco R. Cadaveric nasopharyngeal swab in coronavirus disease 2019 infections: can it be useful for medico-legal purposes? Am J Forensic Med Pathol. 2020;41:238–239. 10.1097/PAF.0000000000000560 32804452

[7] Roberts ISD , Traill ZC. Use of post-mortem computed tomography during the COVID-19 pandemic. Diagn Histopathol. 2021;27:418–421. 10.1016/j.mpdhp.2021.07.002 PMC8318681 34341670

[8] Perdelli F , Spagnolo AM, Cristina ML, et al. Evaluation of contamination by blood aerosols produced during various healthcare procedures. J Hosp Infect. 2008;70:174–179. 18725172 10.1016/j.jhin.2008.06.012

[9] Gerston KF , Blumberg L, Tshabalala VA, et al. Viability of mycobacteria in formalin-fixed lungs. Hum Pathol. 2004;35:571–575. 15138931 10.1016/j.humpath.2004.01.009

[10] Shkrum MJ , Kent J. An autopsy checklist: a monitor of safety and risk management. Am J Forensic Med Pathol. 2016;37:152–157. 27356015 10.1097/PAF.0000000000000244

[11] Li L , Gu J, Shi X, et al. Biosafety level 3 laboratory for autopsies of patients with severe acute respiratory syndrome: principles, practices, and prospects. Clin Infect Dis. 2005;41:815–821. 16107979 10.1086/432720 PMC7107885

[12] Nolte KB , Yoon SS. Theoretical risk for occupational blood-borne infections in forensic pathologists. Infect Control Hosp Epidemiol. 2003;24:772–773. 14587942 10.1086/502131

[13] Burton JL . Health and safety at necropsy. J Clin Pathol. 2003;56:254–260. 12663635 10.1136/jcp.56.4.254 PMC1769932

[14] Lucas SB . HIV and the necropsy. J Clin Pathol. 1993;46:1071–1075. 8282829 10.1136/jcp.46.12.1071 PMC501712

[15] MacArthur S , Schneiderman H. Infection control and the autopsy of persons with human immunodeficiency virus. Am J Infect Control. 1987;15:172–177. 3307544 10.1016/0196-6553(87)90142-8

[16] Abraham JL , Greenfield LJ. Hazard to pathologists and anatomists from vena-caval (Greenfield) filters. Lancet. 1995;346:1100. 10.1016/s0140-6736(95)91773-x 7564808

[17] Weston J , Locker G. Frequency of glove puncture in the post mortem room. J Clin Pathol. 1992;45:177–178. 1541705 10.1136/jcp.45.2.177 PMC495676

[18] Healing TD , Hoffman PN, Young SE. The infection hazards of human cadavers. Commun Dis Rep CDR Rev. 1995;5:R61–68. 7749455

[19] Ruggiero M , Galletti MP, Pacini S, et al. On the risk of contracting AIDS at the dissection table. Ital J Anat Embryol. 2009;114:97–108. 20198822

[20] Ozdemir MH , Aksoy U, Sönmez E, et al. Prevalence of Demodex in health personnel working in the autopsy room. Am J Forensic Med Pathol. 2005;26:18–23. 15725772 10.1097/01.paf.0000154368.04177.fc

[21] Collins CH , Grange JM. Tuberculosis acquired in laboratories and necropsy rooms. Commun Dis Public Health. 1999;2:161–167. 10491868

[22] Claydon SM . The high risk autopsy. Recognition and protection. Am J Forensic Med Pathol. 1993;14:253–256. 8311061 10.1097/00000433-199309000-00016

[23] Li L , Zhang X, Constantine NT, et al. Seroprevalence of parenterally transmitted viruses (HIV-1, HBV, HCV, and HTLV-I/II) in forensic autopsy cases. J Forensic Sci. 1993;38:1075–1083. 8228879

[24] Wilkes MS , Fortin AH, Jacobs TA. Physicians’ attitudes toward the autopsy of patients with AIDS. N Y State J Med. 1991;91:386–389. 1945149

[25] Beaumont LR . The detection of blood on nonporous environmental surfaces: an approach for assessing factors contributing to the risk of occupational exposure to blood in the autopsy suite. Infect Control IC. 1987;8:424–426. 3667120 10.1017/s0195941700066601

[26] Larsh HW , Schwarz J. Accidental inoculation blastomycosis. Cutis. 1977;19:334–335, 337. 844336

[27] Ehrenkranz NJ , Kicklighter JL. Tuberculosis outbreak in a general hospital: evidence for airborne spread of infection. Ann Intern Med. 1972;77:377–382. 5053729 10.7326/0003-4819-77-3-377

[28] Gańczak M , Boroń-Kaczmarska A, Dziuba I. Pathologist and HIV—are safe autopsies possible? Pol J Pathol. 2003;54:143–146. 14575423

[29] Johnson MD , Schaffner W, Atkinson J, et al. Autopsy risk and acquisition of human immunodeficiency virus infection: a case report and reappraisal. Arch Pathol Lab Med. 1997;121:64–66. 9111095

[30] Wilkins D , Woolcock AJ, Cossart YE. Tuberculosis: medical students at risk. Med J Aust. 1994;160:395–397. 8007859

[31] Larson DM , Eckman MR, Alber RL, et al. Primary cutaneous (inoculation) blastomycosis: an occupational hazard to pathologists. Am J Clin Pathol. 1983;79:253–255. 6823911 10.1093/ajcp/79.2.253

[32] Allen RK , Pierson DL, Rodman OG. Cutaneous inoculation tuberculosis: prosector’s wart occurring in a physician. Cutis. 1979;23:815–818. 157262

[33] Stenton SC , Hendrick DJ. Occupational tuberculosis and a failed postgraduate medical examination. Occup Med (Lond). 1996;46:87–88. 8672803 10.1093/occmed/46.1.87

[34] Kantor HS , Poblete R, Pusateri SL. Nosocomial transmission of tuberculosis from unsuspected disease. Am J Med. 1988;84:833–838. 3364442 10.1016/0002-9343(88)90060-5

[35] Lundgren R , Norrman E, Asberg I. Tuberculosis infection transmitted at autopsy. Tubercle. 1987;68:147–150. 3660465 10.1016/0041-3879(87)90032-8

[36] Hawkey PM , Pedler SJ, Southall PJ. Streptococcus pyogenes: a forgotten occupational hazard in the mortuary. BMJ. 1980;281:1058. 7000287 10.1136/bmj.281.6247.1058 PMC1714394

[37] Hooker RP , Eberts TJ, Strickland JA. Primary inoculation tuberculosis. J Hand Surg. 1979;4:270–273. 10.1016/s0363-5023(79)80162-8 155709

[38] Goette DK , Jacobson KW, Doty RD. Primary inoculation tuberculosis of the skin. Prosector’s paronychia. Arch Dermatol. 1978;114:567–569. 646369

[39] Brichacek M , Strazar R, Murray KA, et al. Necrotizing fasciitis after scalpel injury sustained during postmortem examination. CMAJ. 2017;189:E721–23. 28536127 10.1503/cmaj.161386 PMC5436962

[40] Grist NR . Infections in British clinical laboratories 1980–81. J Clin Pathol. 1983;36:121–126. 6826767 10.1136/jcp.36.2.121 PMC498136

[41] Grist NR , Emslie JA. Infections in British clinical laboratories, 1988–1989. J Clin Pathol. 1991;44:667–669. 1890201 10.1136/jcp.44.8.667 PMC496761

[42] Neu HC . Toxoplasmosis transmitted at autopsy. JAMA. 1967;202:844–845. 6072524

[43] Engelen-Lee J-Y , Brouwer MC, Aronica E, et al. Delayed cerebral thrombosis complicating pneumococcal meningitis: an autopsy study. Ann Intensive Care. 2018;8:20. 29427117 10.1186/s13613-018-0368-8 PMC5807251

[44] Pioche C , Pelat C, Larsen C, et al. Estimation de la prévalence de l’hépatite C en population générale, France métropolitaine, 2011. Bull Epidémiol Hebd. 2016;13:224–229. French.

[45] Schleicher S , Schieffer M, Jürgens S, et al. Evidence of multiple hepatitis virus infections in autopsied materials of intravenous drug addicts. Ig Sanita Pubbli. 2005;61:435–450. 17214028

[46] Jänisch S , Günther D, Fieguth A, et al. Post-mortal detection of clostridia—putrefaction or infection? Arch Kriminol. 2010;225:99–108. 20506709

[47] Bush C , Schmid K, Rupp ME, et al. Bloodborne pathogen exposures: difference in reporting rates and individual predictors among health care personnel. Am J Infect Control. 2017;45:372–376. 28063727 10.1016/j.ajic.2016.11.028

[48] Johnson GK , Robinson WS. Human immunodeficiency virus-1 (HIV-1) in the vapors of surgical power instruments. J Med Virol. 1991;33:47–450. 1901908 10.1002/jmv.1890330110

[49] Hutchins KD , Williams AW, Natarajan GA. Neck needle foreign bodies: an added risk for autopsy pathologists. Arch Pathol Lab Med. 2001;125:790–792. 11371232 10.5858/2001-125-0790-NNFB

[50] Miller DC . Creutzfeldt-Jakob disease in histopathology technicians. N Engl J Med. 1988;318:853–854. 3281004 10.1056/NEJM198803313181312

[51] Miller JM , Astles R, Baszler T, et al. Guidelines for safe work practices in human and animal medical diagnostic laboratories. Recommendations of a CDC-convened, biosafety blue ribbon panel. MMWR Suppl. 2012;61:1–102. 22217667

[52] McGuone D , Sinard J, Gill JR, et al. Autopsy services and emergency preparedness of a tertiary academic hospital mortuary for the COVID-19 public health emergency: the Yale plan. Adv Anat Pathol. 2020;27:355–362. 32649315 10.1097/PAP.0000000000000274

